# Evidence that the Ser192Tyr/Arg402Gln in *cis* Tyrosinase gene haplotype is a disease-causing allele in oculocutaneous albinism type 1B (OCA1B)

**DOI:** 10.1038/s41525-021-00275-9

**Published:** 2022-01-13

**Authors:** Siying Lin, Aida Sanchez-Bretaño, Joseph S. Leslie, Katie B. Williams, Helena Lee, N. Simon Thomas, Jonathan Callaway, James Deline, J. Arjuna Ratnayaka, Diana Baralle, Melanie A. Schmitt, Chelsea S. Norman, Sheri Hammond, Gaurav V. Harlalka, Sarah Ennis, Harold E. Cross, Olivia Wenger, Andrew H. Crosby, Emma L. Baple, Jay E. Self

**Affiliations:** 1 RILD Wellcome Wolfson Centre, Royal Devon & Exeter NHS Foundation Trust, Barrack Road, Exeter, UK; 2 Clinical and Experimental Sciences, Faculty of Medicine, University of Southampton, Southampton, UK; 3 Center for Special Children, Vernon Memorial Healthcare, La Farge, WI USA; 4 Southampton Eye Unit, University Hospital Southampton NHS Foundation Trust, Southampton, UK; 5 Faculty of Medicine, University of Southampton, Southampton, UK; 6 Wessex Regional Genetics Laboratory, Salisbury District Hospital, Salisbury, UK; 7 Human Development and Health, Faculty of Medicine, University of Southampton, Southampton, UK; 8 University of Wisconsin School of Medicine and Public Health, Department of Ophthalmology & Visual Sciences, Madison, WI USA; 9 The Rosalind Franklin Institute, Rutherford Appleton Laboratories, Harwell Science and Innovation Campus, Didcot, UK; 10 Rajarshi Shahu College of Pharmacy, Malvihir, Buldana India; 11 Department of Human Genetics and Genomic Medicine, University of Southampton, Southampton, UK; 12 Department of Ophthalmology, University of Arizona College of Medicine, Tucson, AZ USA; 13 New Leaf Clinic, PO Box 336, 16014 East Chestnut Street, Mount Eaton, OH 44691 USA; 14 Department of Pediatrics, Akron Children’s Hospital, 214 West Bowery Street, Akron, OH 44308 USA; 15 Peninsula Clinical Genetics Service, Royal Devon & Exeter Hospital (Heavitree), Gladstone Road, Exeter, UK

**Keywords:** Vision disorders, Genetic testing, Genetics research, Molecular medicine, Genetic testing

## Abstract

Oculocutaneous albinism type 1 (OCA1) is caused by pathogenic variants in the *TYR* (tyrosinase) gene which encodes the critical and rate-limiting enzyme in melanin synthesis. It is the most common OCA subtype found in Caucasians, accounting for ~50% of cases worldwide. The apparent ‘missing heritability’ in OCA is well described, with ~25–30% of clinically diagnosed individuals lacking two clearly pathogenic variants. Here we undertook empowered genetic studies in an extensive multigenerational Amish family, alongside a review of previously published literature, a retrospective analysis of in-house datasets, and tyrosinase activity studies. Together this provides irrefutable evidence of the pathogenicity of two common *TYR* variants, p.(Ser192Tyr) and p.(Arg402Gln) when inherited in *cis* alongside a pathogenic *TYR* variant in *trans*. We also show that homozygosity for the p.(Ser192Tyr)/p.(Arg402Gln) *TYR* haplotype results in a very mild, but fully penetrant, albinism phenotype. Together these data underscore the importance of including the *TYR* p.(Ser192Tyr)/p.(Arg402Gln) in *cis* haplotype as a pathogenic allele causative of OCA, which would likely increase molecular diagnoses in this missing heritability albinism cohort by 25–50%.

## Introduction

Oculocutaneous albinism (OCA) refers to a group of genetically and clinically heterogeneous disorders characterised by abnormal melanin synthesis, resulting in decreased or absent pigmentation of eyes, skin and hair.

Ocular features are present in individuals with OCA and are characteristic of the disease. These include photophobia, nystagmus, foveal hypoplasia, iris transillumination and abnormal decussation of nerve fibres at the optic chiasm resulting in crossed asymmetry on visual evoked potential testing^1^. These ocular features may, however, be variable with no single defining characteristic found to be present in every individual with OCA^2^. The cutaneous phenotype may also vary, ranging from total absence to near-normal levels of pigmentation, and can be difficult to evaluate, particularly in individuals with a lightly pigmented ethnic background^3,4^. As such, OCA can be difficult to distinguish clinically from several other ocular disorders with overlapping phenotypical features, such as *GPR143*-associated X-linked ocular albinism, where the hypopigmentation is limited to the eye^1^, *FRMD7*-associated X-linked idiopathic congenital nystagmus^5^, *SLC38A8*-associated foveal hypoplasia (also known as FHONDA; foveal hypoplasia, optic nerve decussation defects and anterior segment dysgenesis)^6^, and dominant *PAX6*-related ocular developmental disorders^7^.

OCA1, associated with *TYR* gene variants, is the most common OCA subtype found in Caucasians accounting for ~50% of cases worldwide^8,9^. *TYR* encodes the enzyme tyrosinase, which is the critical and rate-limiting enzyme in the biosynthesis of melanin in follicular and epidermal melanocytes in hair and skin, as well as in uveal melanocytes in the iris, ciliary body and choroid, and retinal pigment epithelium cells in the eye^10^. Disease-associated variants in the *TYR* gene cause complete or partial OCA1 depending on their impact on the residual activity of the encoded mutant tyrosinase enzyme^11^. *TYR* gene variants that result in a severe reduction or complete abolition of enzyme activity are associated with OCA1A, characterised by an almost complete absence of hair, skin and eye pigmentation^10,11^. Hypomorphic *TYR* variants in which mutant tyrosinase possess residual catalytic activity are associated with OCA1B, where affected individuals present with a milder phenotype with reduced levels of pigmentation^10,11^.

The apparent missing heritability in OCA is well described, with ~25–30% of clinically affected individuals lacking two clearly pathogenic sequence alterations within the same OCA gene; this proportion is higher in individuals with a partial OCA phenotype^11,12^. Several hypotheses have been proposed to explain this missing heritability, including variants in the promoter or other regulatory elements, as well as epistatic or synergistic interactions between known genes^11,13^. Two *TYR* sequence variants [NM_000372.4:c.575 C > A; **p.(Ser192Tyr)** or S192Y and c.1205 G > A; **p.(Arg402Gln)** or R402Q], previously described as non-pathogenic polymorphisms due to their frequency in the general population (25 and 18% respectively), have been found to be enriched in cohorts of OCA patients with only one identified *TYR* pathogenic variant^8,11,14–22^, leading to suggestions that these variants may in fact account for some of this missing heritability^8,9,14,15,18,23–27^, although this has, however, been disputed by others^17,19,28^. We and others have hypothesised that these variants may be pathogenic only when present in *cis* and inherited in bi-allelic fashion with a second deleterious *TYR* variant for tyrosinase activity to be sufficiently reduced to a level that will cause an OCA phenotype^13,27,29^. However, due to the high frequency of the p.(Ser192Tyr) and p.(Arg402Gln) variants in the general population, and the often small family sizes common to modern European populations, in many cases it has not always been possible to obtain informative allele segregation to phase gene variants and prove inheritance of a *cis* p.(Ser192Tyr)/p.(Arg402Gln) haplotype in *trans* with the pathogenic *TYR* alteration in all affected individuals^27,30^. This remaining uncertainty in clinical interpretation of this haplotype limits its routine reporting in diagnostic testing. This has important diagnostic implications; designating the *TYR* p.(Ser192Tyr)/p.(Arg402Gln) haplotype as pathogenic could substantially increase the diagnostic yield by ~25–50% in albinism patient cohorts with missing heritability^13^. This also further supports the hypothesis that the prevalence of OCA1, commonly quoted as ~1 in 40,000^10^, likely represents a substantial underestimation, particularly amongst Caucasian populations with fair pigmentation^31^. In this study, we present extensive genetic data stemming from our investigation of a large multigenerational extended Amish family, alongside functional studies, a review of genotyped UK based albinism cohorts and a review of existing literature to provide strong evidence to support pathogenicity of the *TYR* p.(Ser192Tyr)/(Arg402Gln) in *cis* haplotype and its contribution to OCA1B in European populations.

## Results

### Clinical findings in an extended Amish family

**Fig. 1:**
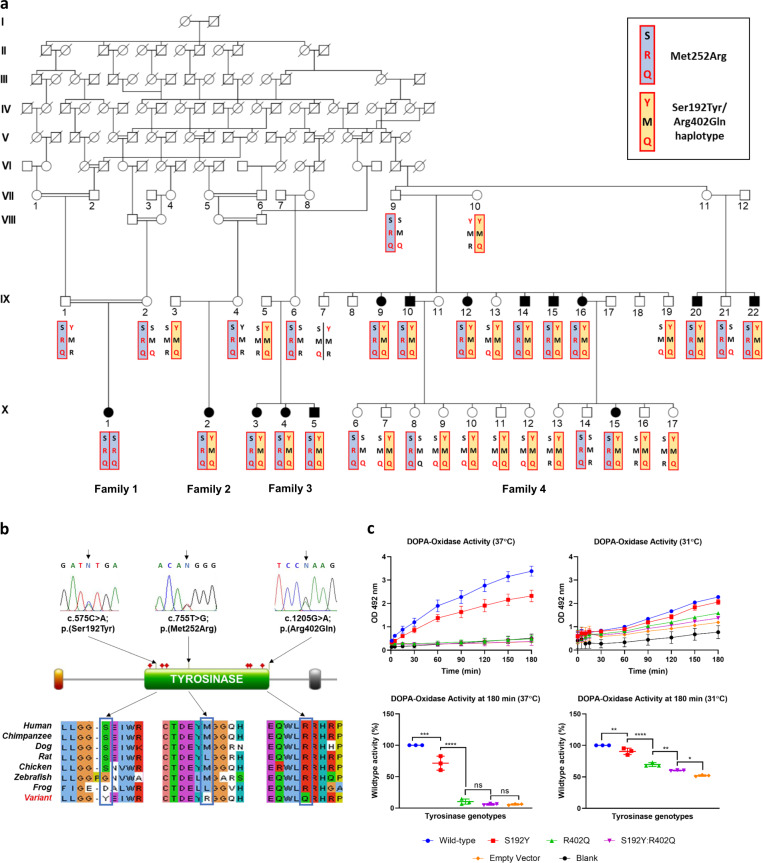
Pedigree diagrams, *TYR* genotype and functional data. **a** Pedigree diagram showing segregation of *TYR* variants p.(Ser192Tyr), p.(Arg402Gln) and p.(Met252Arg) (highlighted in red). The two disease-causing haplotypes are shaded; the p.(Met252Arg) haplotype in blue, and the p.(Ser192Tyr)/p.(Arg402Gln) in *cis* haplotype in yellow. **b** Sequence chromatograms showing *TYR* c.575 C > A; p.(Ser192Tyr), c.755 T > G; p.(Met252Arg) and c.1205 G > A; p.(Arg402Gln) variants in heterozygous form. Schematic localisation of *TYR* p.(Ser192Tyr), p.(Met252Arg) and p.(Arg402Gln) variants within the catalytic tyrosinase domain of the TYR polypeptide. The p.(Ser192Tyr) and p.(Arg402Gln) variants are located at or near the copper-containing catalytic binding sites (the red diamonds denote the histidine residues that bind to copper atoms and hence structurally coordinate the positions of the metal-binding sites). Conservation of *TYR* p.(Ser192Tyr), p.(Met252Arg) and p.(Arg402Gln) variants across species. **c** Tyrosinase activity in wild-type, p.(Ser192Tyr)/S192Y mutant, p.(Arg402Gln)/R402Q mutant and double-mutant HEK293 cells. The absorbance of dopachrome, a product synthesised by the transformation of L-DOPA by tyrosinase was quantified as a measure of tyrosinase activity in wild-type and *TYR*-mutant cell lines. Cumulative production of dopachrome (top row) was quantified from the start of L-DOPA treatment (0 min) to 180 min. Statistical differences between cell lines were analysed at 180 min (bottom row). Data are shown as mean ± SEM and statistically significant differences between groups are indicated by asterisks (*p < 0.05, **p < 0.01, ***p < 0.001, ****p < 0.0001); ns not significant.

We initially investigated a large multigenerational extended Old Order Amish family of Ohio ancestry residing in Wisconsin (USA) with 9 affected individuals all exhibiting nystagmus and variable levels of hair and skin hypopigmentation (Fig. 1; family 4). On the basis of a detailed medical history, assessment of skin and hair pigmentation, and ophthalmic investigations in selected affected individuals, a diagnosis of likely mild OCA was made in all affected individuals. We subsequently recruited two additional Amish families with a total of four affected individuals with a similar clinical phenotype (Fig. 1; families 2 and 3). In addition, a further Amish family with a single affected individual with OCA was recruited to the study (Fig. 1; family 1). This individual displayed clinical features consistent with a complete OCA phenotype, including pale skin and white/blonde hair and eyelashes, nystagmus, iris transillumination defects and foveal hypoplasia. Affected individuals were not noted to bruise or bleed easily, although specific haematological investigations were not performed. Clinical findings for all affected individuals are summarised in Table 1.

**Table 1:** Summary of clinical features observed in affected individuals in families 1–4 with OCA.

Family (ID)	Nystagmus	Hair colour	Eye colour	Other ocular features	Other systemic features
1 (X:1)	+	Blonde	Blue	Iris transillumination defects, depigmented fundus, foveal hypoplasia, alternating esotropia, optic disc hypoplasia	−
2 (X:2)	+	NA	NA	Blunted foveal reflex, depigmented fundus. ERG limited and awaiting a repeat	NA
3 (X:3)	+	Blonde	Blue	Iris transillumination defects blunted foveal reflex	−
3 (X:4)	+	Dark blonde	Blue	transillumination defects, foveal hypoplasia, strabismus	−
3 (X:5)	NA	Strawberry blonde	NA	Blunted foveal reflex	−
4 (IX:9)	+	Blonde	Blue	Pale fundi, iris transillumination defects, foveal hypoplasia, myopia, strabismus. Nyctalopia, photosensitivity and peripheral VF loss with normal ERG	
4 (IX:10)	+	Pigmented	Blue	−	Mild learning difficulties
4 (IX:12)	+	Light brown	NA	−	−
4 (IX:14)	+	Dark brown	Blue	Pale fundi	−
4 (IX:15)	+	Pigmented	Blue	−	−
4 (IX:16)	+	NA	NA	NA	NA
4 (IX:20)	+	Blonde	Blue	−	−
4 (IX:22)	+	White/ blonde	Blue	−	−
4 (X:15)	+	Brown	Brown	Myopia	Neonatal intraventricular haemorrhage

The (+) and (−) symbols indicate the presence or absence of a feature in an affected subject, respectively.

*ERG* electroretinogram, *NA* information not available.

### Genetic findings in Amish families (families 1–4)

Exome sequencing was initially performed in two affected individuals in family 4 (individuals IX:9 and IX:22) for targeted evaluation using the “Albinism or congenital nystagmus v1.0” PanelApp virtual gene panel (41 genes). Subsequently, variants predicted to have a functional consequence (including copy number variants) located genome-wide were identified and filtered according to allele frequency (gnomAD minor allele frequency (MAF) of <0.01). This identified only a single plausible candidate disease variant in both individuals, a heterozygous *TYR* missense variant (GRCh38) chr11:g.89178708 T > G; NM_000372.4:c.755 T > G; p.(Met252Arg) or M252R. The p.Met252 amino acid residue is located in the catalytic domain of the tyrosinase protein and is conserved across a variety of vertebrate species (Fig. 1b). This variant was absent in gnomAD and Genome Project population databases, although it was present in an Amish control exome dataset (allele frequency 0.0023) in heterozygous form only. In silico analysis of the p.(Met252Arg) variant using SIFT, PolyPhen-2 and PROVEAN predicted the variant to be deleterious, possibly damaging and deleterious. This variant has been reported in the compound heterozygous form [with a previously reported p.(Arg217Trp) variant] in a single individual with OCA^22^ and is considered to be likely pathogenic. Exome sequencing did not identify any additional candidate single nucleotide or structural disease variants in any OCA-associated genes.

To explore this apparent missing heritability, targeted dideoxy sequencing of all coding regions and intron-exon junctions of the *TYR* gene was performed in these two individuals. This confirmed the presence of the p.(Met252Arg) variant and also identified a further two *TYR* missense variants (GRCh38) chr11:g.89178528 C > A; NM_000372.4:c.575 C > A; p.(Ser192Tyr) (S192Y) and (GRCh38) chr11:g.89284793 G > A; NM_000372.4:c.1205 G > A; p.(Arg402Gln) (R402Q) in the same two individuals, excluded from the exome sequencing analysis based on individual population frequencies of 0.25 and 0.18, respectively. Segregation of all three *TYR* variants in all Amish families (families 1–4) is shown in Fig. 1, which demonstrates that the p.(Ser192Tyr)/p.(Arg402Gln) variants were linked in *cis* and inherited in a compound heterozygous fashion with p.(Met252Arg) (which itself occurs in *cis* with p.(Arg402Gln)) in all affected individuals except for a single affected individual with OCA, found to be homozygous for p.(Met252Arg) through targeted dideoxy sequencing. Individuals compound heterozygous for *TYR* p.(Met252Arg) and p.(Ser192Tyr)/p.(Arg402Gln) alleles displayed clinical features suggestive of partial albinism with variable skin and hair depigmentation, while the individual homozygous for the *TYR* p.(Met252Arg) variant displayed features of classical OCA including nystagmus, iris transillumination defects, a depigmented fundus and foveal hypoplasia (Table 1). Notably, individuals carrying the *TYR* p.(Met252Arg) variant on one allele and only the p.(Arg402Gln) or the p.(Ser192Tyr) variant on the other allele were apparently unaffected with no clinical features of OCA (individuals VIII:9, IX:2, IX:21, X:6, X:8, IX:1 and IX:4; Fig. 1a).

### Additive temperature-sensitive effects of p.(Ser192Tyr) (S192Y) and p.(Arg402Gln) (R402Q) variants on TYR enzymatic activity

The *TYR* p.(Arg402Gln) variant alone has previously been proposed to contribute to OCA when inherited in *trans* with a pathogenic *TYR* variant^9,14–16,22,23,25,26,32^. Our pedigree analysis, however, appears to dispute this, with five individuals compound heterozygous for the pathogenic *TYR* p.(Met252Arg) variant as well as the p.(Arg402Gln) variant and yet showing no clinical features of OCA (individuals VIII:9, IX:2, IX:21, X:6 and X:8; Fig. 1a). At the same time, 13 individuals who were compound heterozygous for *TYR* p.(Met252Arg) and p.(Ser192Tyr)/p.(Arg402Gln) alleles all displayed clinical features of partial albinism, suggesting an additive impact of the p.(Ser192Tyr) and p.(Arg402Gln) variants on tyrosinase function. To investigate this further, we designed functional experiments to study and quantify the effects of the p.(Ser192Tyr) and p.(Arg402Gln) variants both independently and in combination compared to wild-type tyrosinase enzyme.

Figure 1c shows the DOPA-oxidase activity for all tyrosinase mutants analysed from 0 min to 180 min at 31 °C and 37 °C. At 37 °C, a slight decrease in DOPA-oxidase activity of the p.(Ser192Tyr) mutants was observed, and an almost total loss of DOPA-oxidase activity in the p.(Arg402Gln) mutants and p.(Ser192Tyr)/p.(Arg402Gln) double mutants. At 31 °C, the loss of tyrosinase activity caused by all of the TYR-mutants was reduced but still significant when compared to wildtype. For all the TYR mutant cell lines, the p.(Ser192Tyr)/p.(Arg402Gln) double mutants showed the most reduced tyrosinase activity, followed by p.(Arg402Gln) mutant, with the p.(Ser192Tyr) mutant least affected. There was a statistically significant difference between all three mutant groups, indicative of a cumulative effect of both p.(Ser192Tyr) and p.(Arg402Gln) mutations on tyrosinase activity.

### Enrichment of the *TYR* p.(Ser192Tyr)/p.(Arg402Gln) haplotype in OCA and control cohorts

Interrogation of a clinical cohort of 161 affected individuals with nystagmus and/or albinism (Southampton cohort) (including individuals previously reported by Norman et al. and O’Gorman et al.^13,27^) identified 71 individuals with two pathogenic or likely pathogenic variants (molecularly diagnosed including *TYR*, *OCA2*, *GPR143* and *PAX6* genes), 51 individuals carrying only a single likely disease-associated *TYR* variant with no candidate pathogenic variants identified in other OCA genes (missing heritability), and 39 individuals with no disease-associated *TYR* variants. All patients were sequenced using either the “Albinism or congenital nystagmus v1.0” PanelApp gene panel (41 genes) (https://panelapp.genomicsengland.co.uk/panels/) or a broader research panel as previously described^13,27^. Copy number analysis was not performed. Of these, 2 of the 71 individuals in the molecularly diagnosed group and 49 of the 51 individuals in the missing heritability group were found to have a genotype consistent with the presence of the *TYR* p.(Ser192Tyr)/p.(Arg402Gln) haplotype (i.e. individuals who were homozygous or heterozygous for both these variants) (Table 2); this information was unavailable for the 39 molecularly undiagnosed individuals in this clinical cohort. A review of seven published OCA cohorts with missing heritability (i.e. individuals in whom only a single pathogenic *TYR* variant has been identified), together with our study cohort, found that approximately half of all affected individuals (50.7%) had a genotype consistent with the *TYR* p.(Ser192Tyr)/p.(Arg402Gln) haplotype (Table 2). This is markedly enriched compared to molecularly diagnosed OCA cohorts (2.0%), as well as a control cohort of Amish individuals with no OCA diagnoses (16.9%; Pearson’s Chi-squared test, *p* < 2.2e-16). These findings strongly suggest that the *TYR* p.(Ser192Tyr)/p.(Arg402Gln) haplotype contributes to the OCA phenotype.

**Table 2:** Prevalence of both TYR p.(Ser192Tyr)/S192Y and p.(Arg402Gln)/R402Q variants in OCA cohorts.

	OCA cohorts with missing heritability (individuals with only 1 *TYR* pathogenic or likely pathogenic variant identified)								Molecularly diagnosed OCA1 cohorts (individuals with 2 *TYR* pathogenic or likely pathogenic variant identified)			
	This study^a^	Hutton & Spritz^8^	Hutton & Spritz^15^	Oetting^17^	Ghodsinejad Kalahroudi^20^	Lasseaux^22^	Gronskov^31^	Campbell^30^	Hutton & Spritz^15^	Oetting^17^	Ghodsinejad Kalahroudi^20^	Gronskov^31^
Phenotype	Nystagmus and/or albinism	AROA/ mild OCA	OCA	OCA1	OCA1	Nystagmus and/or absence of fovea	Albinism (OCA, AROA or OA)	Nystagmus and at least one other ocular feature of albinism, no skin hypopigmentation	OCA	OCA1	OCA1	Albinism (OCA, AROA or OA)
Country (ethnicity)	England	(Caucasian)	USA, Canada, Northern Europe (non-Hispanic/ Latino Caucasians)	NA	(Iranian)	France	Scandinavia (Scandinavian)	England	USA, Canada, Northern Europe (non-Hispanic/ Latino Caucasians)	NA	(Iranian)	Scandinavia (Scandinavian)
No of the individuals in the cohort	51	20	13	3	6	158	29	4	71	9	19	2
No of individuals hom or het for both *TYR* S192Y & R402Q	49	1	3	2	0	64	21	4	2	0	0	0
Proportion of study cohort where S192Y/R402Q haplotype is possible	49/51 (96.1%)	1/20 (5%)	3/13 (23.1%)	2/3 (66.7%)	0/6 (0%)	64/158 (40.5%)	21/29 (72.4%)	4/4 (100%)	2/71 (2.8%)	0/9 (0%)	0/19 (0%)	0/2 (0%)
Combined proportion where S192Y/R402Q haplotype is possible					144/284 (50.7%)					2/101 (2.0%)		

*AROA* autosomal recessive ocular albinism, *AXD* in-house Amish exome database, *het* heterozygous, *hom* homozygous, *OCA* oculocutaneous albinism, *OA* ocular albinism, *no* number.

^a^This cohort includes individuals previously reported in Norman et al. and O’Gorman et al.

### Prevalence of *TYR* p.(Ser192Tyr)/p.(Arg402Gln) haplotype in OCA cohorts with missing heritability

Forty-nine affected individuals in our (Southampton and Salisbury) study cohorts were identified as carrying only a single pathogenic or likely pathogenic *TYR* variant as well as harbouring homozygous or heterozygous *TYR* p.(Ser192Tyr) and p.(Arg402Gln) variants; of these, familial segregation was performed in 41 individuals and their parents to assess the phase of the variants. In 23 individuals, this confirmed that the *TYR* p.(Ser192Tyr) and p.(Arg402Gln) variants were inherited in *cis*, and this haplotype was in *trans* to the previously identified pathogenic or likely pathogenic *TYR* variant (Table 3). For the remaining 18 cases, definitive segregation was not possible. Notably, no case was identified in which segregation showed that p.(Ser192Tyr) and p.(Arg402Gln) were not in *trans* with the pathogenic or likely pathogenic variant.

**Table 3:** Potential contribution of *TYR* S192Y/R402Q haplotype to molecular diagnoses in OCA cohorts with missing heritability.

Study	This study^a^	Oetting^17^	Ghodsinejad Kalahroudi^20^	Lasseaux^22^	Gronskov^31^	Campbell^30^
Phenotype	Nystagmus and/or albinism	OCA1	OCA1	Nystagmus and/or absence of fovea	Albinism (OCA, AROA or OA)	Nystagmus and at least one other ocular feature of albinism, no skin hypopigmentation
Country (ethnicity)	England	NA	(Iranian)	France	Scandinavia (Scandinavian)	England
Number of individuals in the cohort	51	3	6	158	29	4
Number of individuals hom or het for both *TYR* S192Y and R402Q, where S192Y/R402Q haplotype is possible	49	2	0	64	21	4
Number of individuals in whom it was possible to determine the phase of *TYR* S192Y, R402Q and pathogenic or likely pathogenic variants (“informative cohort”)	23	2	6	31	6	2
Number of individuals in whom *TYR* S192Y and R402Q were in *cis*, and in *trans* to pathogenic or likely pathogenic *TYR* variant in the informative cohort	23	2	0	31	6	2
The proportion of “informative cohort” where S192Y/R402Q haplotype is possible and molecular diagnoses due to *TYR* S192Y/R402Q haplotype in *trans* to pathogenic or likely pathogenic *TYR* variant	23/23 (100%)	2/2 (100%)	S192Y/R402Q haplotype not possible in any individuals in the study	31/31 (100%)	6/6 (100%)	2/2 (100%)
Combined proportion of “informative cohort” where molecular diagnoses are due to *TYR* S192Y/R402Q haplotype in *trans* to pathogenic or likely pathogenic *TYR* variant			64/64 (100%)			
The proportion of cohort where molecular diagnoses due to *TYR* S192Y/R402Q haplotype in *trans* to pathogenic or likely pathogenic *TYR* variant	23/51 (45.1%)	2/3 (66.7%)	0/6 (0%)	31/158 (19.6%)	6/29 (20.7%)	2/4 (50%)
Combined proportion of cohort where molecular diagnoses are due to *TYR* S192Y/R402Q haplotype in *trans* to pathogenic or likely pathogenic *TYR* variant			64/251 (25.5%)			

This includes individuals with only 1 *TYR* pathogenic or likely pathogenic variant identified.

*AROA* autosomal recessive ocular albinism, *het* heterozygous, *hom* homozygous, *OCA* oculocutaneous albinism, *OA* ocular albinism.

^a^This cohort includes individuals previously reported in Norman et al. and O’Gorman et al.

In five of the seven published OCA cohorts with missing heritability reviewed (Table 2), it was possible to determine the *cis/trans* phase of the *TYR* p.(Ser192Tyr) and p.(Arg402Gln) variants in a proportion of individuals reported^17,20,22,30,31^ (Table 3); in the remaining individuals this was not possible due to familial samples being unavailable for segregation analysis, or uninformative segregation results (owing to the high allele frequency of the p.(Ser192Tyr) and p.(Arg402Gln) *TYR* variants in the general population). For the remaining two studies of OCA cohorts with missing heritability, the *cis/trans* phase of the *TYR* p.(Ser192Tyr) and p.(Arg402Gln) variants could not be determined from the reported genotypes^8,15^. There were 41 OCA individuals with missing heritability from these five studies in whom the p.(Ser192Tyr)/p.(Arg402Gln) haplotype was possible, and where the *cis/trans* phase of the *TYR* p.(Ser192Tyr) and p.(Arg402Gln) variants could also be determined. In accordance with the findings from our local research cohorts, together with this additional informative cohort derived from five published studies, the *TYR* p.(Ser192Tyr)/p.(Arg402Gln) haplotype segregated in *trans* with the pathogenic *TYR* variant in all 64 cases (amounting to 25.5% of total missing heritability cases) (Table 3). Taken together with the findings in Table 2, this suggests that the p.(Ser192Tyr)/p.(Arg402Gln) haplotype completes the molecular diagnosis in ~25–50% of OCA individuals with missing heritability.

## Discussion

The pathogenicity of *TYR* p.(Ser192Tyr) and p.(Arg402Gln) variants and their contribution to the OCA phenotype, either in isolation or when linked in *cis*, has been heavily debated in many studies^8,9,14,15,17–19,23–28^. As such, these *TYR* variants are variably reported by clinical testing laboratories and potentially excluded, even when shown to be in *cis*. Here our genomic and functional data, initiated by our search for the cause of OCA in a number of Amish families, provide irrefutably strong evidence that the *TYR* p.(Ser192Tyr) and p.(Arg402Gln) variants are pathogenic when in *cis*. The increased frequency of the *TYR* p.(Met252Arg) variant in the Amish community, likely due to founder effects and endogamy, together with the large family sizes typical within the community, permitted empowered cosegregation studies able to determine the haplotype, phasing and inheritance of the common p.(Ser192Tyr) and p.(Arg402Gln) *TYR* variants together with the p.(Met252Arg) variant in a large number of related individuals.

Both Jagirdar et al. and our group have previously proposed that both *TYR* p.(Ser192Tyr) and p.(Arg402Gln) variants, acting in *cis*, may have an additive effect producing a greater reduction in enzyme activity compared to each variant individually^27,29^. Both p.(Ser192Tyr) and p.(Arg402Gln) variants are common in Caucasian populations with allele frequencies of 36% and 27%, respectively (gnomAD v2.1.1), and would thus normally be considered benign. Indeed, our study demonstrates that inheritance of either variant individually in compound heterozygous form with the deleterious p.(Met252Arg) variant is insufficient to result in an OCA phenotype (individuals VIII:9, IX:1, IX:2, IX:4, X:6 and X:8; Fig. 1a). The p.(Ser192Tyr) and p.(Arg402Gln) variants are believed to have arisen independently on different ancestral haplotypes^33^, and their combined presence in *cis* on a recombinant haplotype is relatively rare, predicted to be between 1.1% to 1.9% in European populations^27,29,31^. Our studies here, alongside other previous studies^8,13,17,22,27,30,31^, provide strong support to show that the *TYR* p.(Ser192Tyr)/p.(Arg402Gln) the haplotype is enriched in Caucasian OCA cohorts with missing heritability (Table 2), and contributes to an OCA1B diagnosis when inherited in *trans* with a second deleterious *TYR* variant, particularly in individuals with lower pigmentary backgrounds, who may be more susceptible to the damaging effects of hypomorphic variants^16,34^. However, given the number of apparently unaffected individuals homozygous for the p.(Ser192Tyr)/p.(Arg402Gln) haplotype reported in the literature (Supplementary Table 1)^29–31^, the penetrance of the p.(Ser192Tyr)/p.(Arg402Gln) haplotype might appear to be incomplete, confounding the argument that it is a pathogenic allele. The apparently reduced penetrance of the *TYR* p.(Ser192Tyr)/p.(Arg402Gln) haplotype may relate to the modifying effects of sequence variants in genes encoding other melanosomal proteins^35–39^, although other genetic and molecular studies would be required to confirm this. However, we propose that individuals homozygous for the hypomorphic *TYR* p.(Ser192Tyr)/p.(Arg402Gln) allele may have instead a consistent but mild phenotype, which is easily missed by incomplete phenotyping. In support of this, our studies identified five individuals with a clinical diagnosis of ‘possible hypomorphic’ OCA who were homozygous for *TYR* p.(Ser192Tyr)/p.(Arg402Gln), with no other known or likely *TYR* or other OCA gene-associated variants identified (Supplementary Table 1). All were noted to have foveal hypoplasia on OCT investigation but most had very mild, if any, other OCA features. Additionally, an apparently unaffected relative in our study was also identified as homozygous for *TYR* p.(Ser192Tyr)/p.(Arg402Gln). Despite the absence of nystagmus or any other pigmentary phenotype in this unaffected individual and visual acuities of 0.1 and 0.08 LogMAR (right and left eye respectively), the further detailed clinical investigation identified very mild iris transillumination and significant foveal hypoplasia (Supplementary Fig. 1). A review of the literature identified a further seven affected individuals in two studies with detailed clinical phenotyping available^30,31^ (Supplementary Table 1). Foveal hypoplasia, as well as iris transillumination, was documented in all thirteen individuals homozygous for both *TYR* p.(Ser192Tyr) and p.(Arg402Gln) (Supplementary Table 1). It, therefore, seems possible that individuals homozygous for the hypomorphic p.(Ser192Tyr)/p.(Arg402Gln) *TYR* allele have such a mild phenotype that they can easily go unidentified and unreported due to minimal effects on visual function or clear features of albinism; further phenotypic studies in large genomic population cohorts may be able to further clarify this potential association.

The *TYR* p.(Arg402Gln) variant is located near the copper-containing catalytic binding site CuB, and functional studies have shown that this amino acid alteration results in an enzyme with decreased thermal stability, disrupted copper-binding and reduced catalytic activity, thought to be mediated by decreased protein stability resulting in increased retention of the mutant tyrosinase protein as an unprocessed and misfolded glycoform in the endoplasmic reticulum (ER)^29,40–46^. The *TYR* p.(Ser192Tyr) variant is located within the copper-containing catalytic binding site CuA, and has been shown to reduce tyrosinase enzymatic activity and melanocyte pigment production independent of the p.(Arg402Gln) variant^29,47,48^. Genome-wide association studies have identified associations with skin, hair and eye pigmentation for both p.(Ser192Tyr) and p.(Arg402Gln) variants^49–53^, suggesting these *TYR* variants have a role in normal pigmentary variation, and that the double-variant p.(Ser192Tyr)/p.(Arg402Gln) haplotype appears to show an additive effect on these pigmentary phenotypes compared to each variant individually^29^. It is difficult however from the literature review alone to quantify the functional effects of the p.(Ser192Tyr) and p.(Arg402Gln) *TYR* variants both independently and in combination, compared to wild-type tyrosinase enzyme. This issue arises from the historical use of the human *TYR* expression construct pcTYR containing the p.(Ser192Tyr) variant to study the effects of “wildtype” tyrosinase activity^42,54^. Computational approaches to TYR functional activity, based on protein flexibility and dynamic properties, suggest that the p.(Ser192Tyr) and p.(Arg402Gln) variants both result in a TYR protein that is less stable and has reduced enzyme activity compared to a wild-type molecule; the combined effect of having both changes together in a single TYR molecule, however, has not been previously investigated^55^. Our study now shows for the first time a thermosensitive additive decrease in enzymatic function of the double-variant p.(Ser192Tyr)/p.(Arg402Gln) TYR protein compared to each variant acting individually (Fig. 1c), lending further support to the pathogenicity of the p.(Ser192Tyr)/p.(Arg402Gln) haplotype. Homology modelling of tyrosinase protein structure does not appear to show a direct interaction between the 192 and 402 amino acid residues^31^, and therefore this additional reduction in enzyme function in the double-mutant protein may instead be mediated by a combination of increased ER retention of the misfolded mutant protein [caused by p.(Arg402Gln) reducing protein stability] and reduced enzyme activity of any released mutant protein [possibly resulting from steric hindrance effects of p.(Ser192Tyr) affecting the CuA binding site]^47^, as proposed by Gronskov et al.^31^.

Subcellular localisation studies have determined that disease-associated *TYR* variants commonly result in near-absolute and irreversible ER retention of the mutant protein. The p.(Arg402Gln) variant, however, results in a thermosensitive tyrosinase protein that is retained in the ER at higher temperatures but is able to partially exit the ER at lower, more permissive temperatures^40,44,46^. Homozygosity for the p.(Ser192Tyr)/p.(Arg402Gln) haplotype may therefore still permit sufficient quantities of mutant tyrosinase to reach the inner surface of the melanosomal membrane, where the mutant protein is still able to participate in protein–protein interactions with other melanosomal proteins involved in melanogenesis, such as TYRP1 and TYRP2^56,57^, resulting in a less severe functional impact and a milder pigmentary phenotype that may not always be clinically significant. This thermosensitivity of the double-variant mutant TYR protein also provides a compelling explanation for our discovery of a consistent foveal hypoplasia phenotype in individuals who are homozygous for both p.(Ser192Tyr)/p.(Arg402Gln) *TYR* variants, as higher temperatures within the developing eye may result in a larger impact of these variants on tyrosinase function^14^, while lower temperatures at the skin and extremities instead result in greater preservation of mutant protein function and a milder and more variable pigmentary phenotype.

Together, our studies define the genotype, biochemical and phenotype correlation of the p.(Met252Arg) and p.(Ser192Tyr)/p.(Arg402Gln) *TYR* variants and collectively demonstrate that the in *cis* p.(Ser192Tyr)/p.(Arg402Gln) allele is pathogenic. As such, the *TYR* p.(Ser192Tyr)/p.(Arg402Gln) haplotype should be included as a pathogenic allele in future and retrospective genetic diagnoses of OCA, supporting the idea for a review of all previously undiagnosed OCA cases where these variants have been excluded. Reporting of the p.(Ser192Tyr)/p.(Arg402Gln) genotype in individuals in whom only a single deleterious *TYR* variant has been identified could permit a 25–50% uplift in confirmatory molecular diagnoses (when the phase has been determined) in this diagnostically challenging patient group (Tables 2, 3). Additionally, for patients with an albinism phenotype but no apparent variants in albinism genes, consideration of these variants when identified in *cis* as a pathogenic allele in its own right may also help provide clinical direction. For example, in individuals heterozygous for this allele, alternative diagnoses such as syndromic albinism might be considered less likely as they would be considered ‘at least a carrier of a pathogenic OCA1B allele’, and genomic data may be re-examined in a targeted fashion to search for further non-coding splice or structural variants in the *TYR* gene. In individuals with a very mild albinism phenotype or isolated foveal hypoplasia, identification of this pathogenic allele in homozygous form may provide the molecular diagnosis, ending their diagnostic odyssey. It will be crucially important to accurately determine the phase of these common variants, and due to the high frequencies of these variants alone in the population which can limit informative phase studies in relatives, consideration should perhaps be given to the use of amplicon-based long-read sequencing technologies that allow haplotype phasing in the genomic workup of such patients^58^. Achieving an accurate molecular diagnosis will bring about important benefits in affected individuals and their families, allowing accurate prognostic information and family counselling to be provided, avoiding the need for further invasive investigations to confirm the clinical diagnosis or rule out syndromic forms of the disease or masquerading conditions, and has important therapeutic implications, given the emerging therapies currently under development and in clinical trials for OCA^59,60^.

## Methods

### Ethics statement

This study was approved by the institutional review board of all participating institutions (University of Arizona IRB—1000000050, Akron Children’s Hospital IRB—project number 986876–3, South Central—Hampshire A Research Ethics Committee—IRAS:174564), and all participating individuals were recruited with written informed consent.

### Patient ascertainment and clinical phenotyping

Affected individuals and unaffected family members from four Ohio and Wisconsin Amish families with a common Ohio ancestry were recruited to this study (Fig. 1). Medical history was taken in all recruited family members, as well as detailed phenotyping of skin and hair pigmentation, particularly in the context of familial pigmentary background. A diagnosis of nystagmus was established in all affected individuals, and further ophthalmic investigations including electroretinography and optical coherence tomography (OCT) were performed in selected individuals. Blood/buccal samples were obtained with informed consent.

### Molecular genetic analysis

Participating individuals had either peripheral venous blood samples taken in EDTA containing vacutainer tubes or buccal cell collection using the ORAcollect® for paediatrics kit (DNA Genotek). Genomic DNA extraction was performed using either the ReliaPrep^TM^ kit (Blood gDNA Miniprep System, Promega) for venous blood samples or the Xtreme DNA kit (Isohelix) for buccal samples, according to the manufacturer’s protocol. Exome sequencing (whole-exome sequencing, Exeter laboratory for individual IX:9 and Illumina TruSight^TM^ One clinical exome sequencing panel, Southampton laboratory for individual IX:22) was performed as previously described^27,61^. The whole-exome sequencing sample was prepared using Agilent Sureselect Whole Exome v6 targeting, while the TruSight^TM^ One panel provides targeted sequencing for 4813 genes associated with clinical phenotypes and captures most of the coding regions of genes responsible for OCA subtypes 1–4 & 6 (*TYR*, *OCA2*, *TYRP1*, *SLC45A2* and *SLC24A5*, respectively), the ocular albinism gene (*GPR143*), all syndromic albinism genes and *PAX6*. Next-generation sequencing analysis (NextSeq500: Illumina) involved: read alignment (BWA-MEM (v0.7.12), mate-pairs fixed and duplicates removed (Picard v1.129), InDel realignment/base quality recalibration (GATK v3.4–46), single-nucleotide variant (SNV)/InDel detection (GATK HaplotypeCaller), annotation (Alamut v1.4.4), and read depth (GATK DepthOfCoverage). Additional filtering was performed using virtual gene panel analysis of exome data using the “Albinism or congenital nystagmus v1.0” PanelApp gene panel (41 genes) (https://panelapp.genomicsengland.co.uk/panels/), with variants prioritised by call quality, frequency in control datasets (Genome Aggregation Database; gnomAD v2.1.1 and 1000 Genomes Project) and predicted functional consequence^13,27^. Primers were designed with Primer3 web software to cover all five coding exons and associated intron-exon junctions in *TYR*. As the 3′ region encompassing coding exons 4 and 5 of *TYR* shares high homology with a pseudogene, *TYRL*^62^, locus-specific amplification primers were designed for *TYR* exons 4 and 5 to prevent co-amplification of *TYR* and *TYRL* and subsequent misinterpretation of results. Dideoxy sequencing products were sequenced by Source BioScience Lifesciences (https://www.sourcebioscience.com/). Primer sequences and polymerase chain reaction conditions are listed in supplementary Table 3. The *TYR* c.755 T > G; p.(Met252Arg) variant and c.[575 C > A;1205 G > A]; p.[Ser192Tyr;Arg402Gln] variants-in-*cis* haplotype were submitted to ClinVar (www.ncbi.nlm.nih.gov/clinvar, accession numbers SCV001984755 and SCV001984756).

### Establishment of Tyr mutant cell lines

The plasmid vector p3XFLAG-CMV-14 containing *TYR* cDNA was purchased from Addgene (Massachusetts, USA) and was initially deposited by Ruth Halaban^63^. Upon arrival, sequencing revealed the p.(Ser192Tyr) (c.C575A) common population variant to be present. Site-directed mutagenesis was used to create the wild-type sequence (c.575 C, p.192Ser) as well as the p.(Arg402Gln) variant. The primers used for each variant inserted through site-directed mutagenesis are listed in Supplementary Table 2. Site-directed mutagenesis was carried out using the non-strand displacing activity of Pfu DNA polymerase to incorporate and extend the mutagenic primers. The reaction mixture contained Phusion Pfu Polymerase and its buffer, forward and reverse primers (0.5 µM), dNTPs (200 µM) and the cDNA template. PCRs were performed in a total volume of 50 µl. Touch-down PCR conditions were set at 98 °C for 30 sec followed by 30 cycles of 98 °C for 10 sec, 45–72 °C for 10–30 sec and 72 °C for 15–30 sec, and a final extension step of 72 °C for 5–10 min. The PCR product was treated with DpnI to digest the methylated parental DNA.

Purified mutated tyrosinase PCR products were employed to transform NEB® 5-alpha Competent E. coli (High Efficiency; New England Biolabs, UK) via heat shock method. Briefly, 50 µl of thawed cells were kept on ice and combined with ~100 ng of plasmid DNA and incubated for 30 min. The cell-DNA mixture was heat-shocked at 42 °C for 30 sec and then placed on ice for 5 min. Cells were given S.O.C medium and incubated for an hour in a shaking incubator before being plated on ampicillin selection (100 ug/ml) LB agar plates. After overnight incubation at 37 °C, single ampicillin-resistant colonies were picked and grown in LB broth for approximately 16 h, at which point the cells were pelleted by centrifugation and the DNA extracted. When the stocks were diminished, competent cells were produced through treatment with CaCl_2_ and subsequently transformed using the heat shock method described above.

### Cell culture conditions

Human Embryonic Kidney 293 Freestyle (HEK293F) cells (Invitrogen, California, USA) were cultured in Freestyle culture medium (Invitrogen, California, USA) at 37 °C in a shaking incubator at 125 rpm with 8% CO_2_. When cells reached a density of 1 × 10^6^ cells/ml, they were transfected with 30 µg of plasmids containing the p.(Arg402Gln) or p.(Ser192Tyr) mutations or co-transfected with both plasmids. The lipid-based reagent, 293fectin (60 µl) (ThermoFisher, UK), was diluted in Opti-MEM (ThermoFisher, UK) and incubated at room temperature for 5 mins. DNA and 293fectin were combined, gently mixed and incubated at room temperature for 30 mins before adding to cells. Then, cells were incubated in 6 wells plates for 72 h at 31 °C or 37 °C to reach 90% confluency, and the enzymatic activity assays were performed.

### Enzymatic activity assays

The DOPA-oxidase activity was assessed in the different mutants. First, transfected cells from the different mutant clones were treated with L-DOPA, and the DOPA-oxidase activity was measured as the accumulation of the downstream product, dopachrome, following the manufacturer’s protocol. Briefly, cells cultured in six-well plates were lysed in NP40 Cell Lysis Buffer (ThermoFisher, UK) containing 1 mM phenylmethylsulfonyl fluoride (PMSF) (in DMSO with a final concentration of 1%) and 1X protease and phosphatase inhibitor (Halt™ Phosphatase Inhibitor Cocktail, Thermo Fisher Scientific, UK), and protein concentration was measured by BCA assay (Thermo Scientific™ Pierce™ BCA Protein Assay Kit). Samples were then diluted into 4 µg/µl, and 50 µl or 30 µl sample aliquots were used for the DOPA assays. After adding the volume of the samples to 96-well plates, 150 µl of a phosphate buffer with L-DOPA 1 mM was added to the wells. Enzymatic activity was recorded as the absorbance of dopachrome at 492 nm from the start of L-dopa treatment (0 min) and at 30 min intervals thereafter for a total of 180 min at both 31 °C and 37 °C. Assays were routinely performed in triplicate and the results are presented as the means of the independent assays ± standard error.

### Statistics

Results of enzymatic activity at 180 min were normalised to wild-type, with the values for wild-type taken to be 100% of the expected enzymatic activity. One-way ANOVA was performed followed by a Sidak’s post-hoc test. A probability level of at least *p* < 0.05 was considered statistically significant (*p < 0.05, **p < 0.01, ***p < 0.001, ****p < 0.0001).

### Evaluating the prevalence of *TYR* p.(Ser192Tyr)/p.(Arg402Gln) haplotype in OCA and control cohorts

A clinical cohort of affected individuals with nystagmus and/or albinism was retrospectively ascertained through the Southampton (161 individuals) and Salisbury (131 individuals) research databases. All individuals had been referred from a regional paediatric nystagmus clinic. Next-generation sequencing (Illumina TruSight One clinical exome sequencing panel), alignment and filtering were performed as previously described^13,27^. The genomic data were interrogated to ascertain the frequency of the *TYR* p.(Ser192Tyr)/p.(Arg402Gln) haplotype in this cohort. A literature review was also performed to evaluate the reported prevalence of the *TYR* p.(Ser192Tyr)/p.(Arg402Gln) haplotype in additional published OCA cohorts. This was compared against an in-house exome database of Amish individuals unaffected by OCA. Statistical analysis was performed using an established software package (R Core Team 2015; R Foundation for Statistical Computing, Vienna, Austria)^64^.

### Reporting summary

Further information on research design is available in the Nature Research Reporting Summary linked to this article.

## Supplementary information

Supplementary Information

Reporting Summary

## Data Availability

The genetic variants investigated are deposited in ClinVar (accession codes SCV001984755 and SCV001984756). While the in-house Amish exome database is not publicly accessible due to the informed consent restrictions, de-identified information may be accessible and requested from corresponding authors A.H.C. (a.h.crosby@exeter.ac.uk) and E.L.B. (E.Baple@exeter.ac.uk).

## References

[1] McKay BS (2019). Pigmentation and vision: is GPR143 in control?. J. Neurosci. Res..

[2] Kruijt CC (2018). The phenotypic spectrum of albinism. Ophthalmology.

[3] Sjöström A, Kraemer M, Ohlsson J, Villarreal G (2001). Subnormal visual acuity syndromes (SVAS): albinism in Swedish 12-13-year-old children. Doc. Ophthalmol..

[4] Sjöström A (2004). Subnormal visual acuity (svas) and albinism in mexican 12–13-year-old children. Doc. Ophthalmol..

[5] Thomas, M. G., Maconachie, G., Hisaund, M. & Gottlob, I. FRMD7-related infantile nystagmus. *GeneReviews*. https://www.ncbi.nlm.nih.gov/books/NBK3822/ (2009).

[6] Poulter JA (2013). Recessive mutations in SLC38A8 cause foveal hypoplasia and optic nerve misrouting without albinism. Am. J. Hum. Genet..

[7] Lima Cunha, D., Arno, G., Corton, M. & Moosajee, M. The spectrum of PAX6 mutations and genotype-phenotype correlations in the eye. *Genes (Basel)*10.3390/genes10121050 (2019). 10.3390/genes10121050 PMC6947179 31861090

[8] Hutton SM, Spritz RA (2008). Comprehensive analysis of oculocutaneous albinism among non-Hispanic caucasians shows that OCA1 is the most prevalent OCA type. J. Invest. Dermatol..

[9] Rooryck C (2008). Molecular diagnosis of oculocutaneous albinism: new mutations in the OCA1-4 genes and practical aspects. Pigment Cell Melanoma Res.

[10] Grønskov K, Ek J, Brondum-Nielsen K (2007). Oculocutaneous albinism. Orphanet J. Rare Dis..

[11] Simeonov DR (2013). DNA variations in oculocutaneous albinism: an updated mutation list and current outstanding issues in molecular diagnostics. Hum. Mutat..

[12] King RA (2003). Tyrosinase gene mutations in oculocutaneous albinism 1 (OCA1): definition of the phenotype. Hum. Genet.

[13] O’Gorman L (2019). A small gene sequencing panel realises a high diagnostic rate in patients with congenital nystagmus following basic phenotyping. Sci. Rep..

[14] Fukai K (1995). Autosomal recessive ocular albinism associated with a functionally significant tyrosinase gene polymorphism. Nat. Genet..

[15] Hutton SM, Spritz RA (2008). A comprehensive genetic study of autosomal recessive ocular albinism in Caucasian patients. Invest. Ophthalmol. Vis. Sci..

[16] Chiang PW, Spector E, Tsai AC (2009). Oculocutaneous albinism spectrum. Am. J. Med. Genet. A.

[17] Oetting WS (2009). The R402Q tyrosinase variant does not cause autosomal recessive ocular albinism. Am. J. Med. Genet. A.

[18] Gargiulo A (2011). Molecular and clinical characterization of albinism in a large cohort of Italian patients. Invest. Ophthalmol. Vis. Sci..

[19] Preising MN, Forster H, Gonser M, Lorenz B (2011). Screening of TYR, OCA2, GPR143, and MC1R in patients with congenital nystagmus, macular hypoplasia, and fundus hypopigmentation indicating albinism. Mol. Vis..

[20] Ghodsinejad Kalahroudi V (2014). Two novel tyrosinase (TYR) gene mutations with pathogenic impact on oculocutaneous albinism type 1 (OCA1). PLoS ONE.

[21] Mauri L (2017). Clinical evaluation and molecular screening of a large consecutive series of albino patients. J. Hum. Genet.

[22] Lasseaux E (2018). Molecular characterization of a series of 990 index patients with albinism. Pigment Cell Melanoma Res..

[23] Chiang P-W, Drautz JM, Tsai AC-H, Spector E, Clericuzio CL (2008). A new hypothesis of OCA1B. Am. J. Med. Genet. Part A.

[24] Kausar T, Bhatti MA, Ali M, Shaikh RS, Ahmed ZM (2013). OCA5, a novel locus for non-syndromic oculocutaneous albinism, maps to chromosome 4q24. Clin. Genet..

[25] Kubal A, Dagnelie G, Goldberg M (2009). Ocular albinism with absent foveal pits but without nystagmus, photophobia, or severely reduced vision. J. AAPOS.

[26] Thomas MG, Maconachie GD, Sheth V, McLean RJ, Gottlob I (2017). Development and clinical utility of a novel diagnostic nystagmus gene panel using targeted next-generation sequencing. Eur. J. Hum. Genet..

[27] Norman CS (2017). Identification of a functionally significant tri-allelic genotype in the Tyrosinase gene (TYR) causing hypomorphic oculocutaneous albinism (OCA1B). Sci. Rep..

[28] Gronskov K (2009). Birth prevalence and mutation spectrum in danish patients with autosomal recessive albinism. Invest. Ophthalmol. Vis. Sci..

[29] Jagirdar K (2014). Molecular analysis of common polymorphisms within the human Tyrosinase locus and genetic association with pigmentation traits. Pigment Cell Melanoma Res.

[30] Campbell P (2019). Clinical and genetic variability in children with partial albinism. Sci. Rep..

[31] Gronskov K (2019). A pathogenic haplotype, common in Europeans, causes autosomal recessive albinism and uncovers missing heritability in OCA1. Sci. Rep..

[32] Marti A (2018). Lessons of a day hospital: comprehensive assessment of patients with albinism in a European setting. Pigment Cell Melanoma Res..

[33] Hudjashov G, Villems R, Kivisild T (2013). Global patterns of diversity and selection in human tyrosinase gene. PLoS ONE.

[34] Mondal M, Sengupta M, Ray K (2016). Functional assessment of tyrosinase variants identified in individuals with albinism is essential for unequivocal determination of genotype-to-phenotype correlation. Br. J. Dermatol..

[35] King RA (2003). MC1R mutations modify the classic phenotype of oculocutaneous albinism type 2 (OCA2). Am. J. Hum. Genet..

[36] Manga P (1997). Rufous oculocutaneous albinism in southern African Blacks is caused by mutations in the TYRP1 gene. Am. J. Hum. Genet..

[37] Chiang PW, Fulton AB, Spector E, Hisama FM (2008). Synergistic interaction of the OCA2 and OCA3 genes in a family. Am. J. Med. Genet. A.

[38] Sajid, Z. et al. Genetic causes of oculocutaneous albinism in Pakistani population. *Genes (Basel)*10.3390/genes12040492 (2021). 10.3390/genes12040492 PMC8066997 33800529

[39] Wei AH, Yang XM, Lian S, Li W (2013). Genetic analyses of Chinese patients with digenic oculocutaneous albinism. Chin. Med. J. (Engl.).

[40] Berson JF, Frank DW, Calvo PA, Bieler BM, Marks MS (2000). A common temperature-sensitive allelic form of human tyrosinase is retained in the endoplasmic reticulum at the nonpermissive temperature. J. Biol. Chem..

[41] Spritz RA, Ho L, Furumura M, Hearing VJ (1997). Mutational analysis of copper binding by human tyrosinase. J. Invest. Dermatol..

[42] Tripathi RK, Giebel LB, Strunk KM, Spritz RA (1991). A polymorphism of the human tyrosinase gene is associated with temperature-sensitive enzymatic activity. Gene Expr..

[43] Tripathi RK, Hearing VJ, Urabe K, Aroca P, Spritz RA (1992). Mutational mapping of the catalytic activities of human tyrosinase. J. Biol. Chem..

[44] Halaban R (2000). Endoplasmic reticulum retention is a common defect associated with tyrosinase-negative albinism. Proc. Natl Acad. Sci. USA.

[45] Dolinska MB (2017). Oculocutaneous albinism type 1: link between mutations, tyrosinase conformational stability, and enzymatic activity. Pigment Cell Melanoma Res..

[46] Toyofuku K, Wada I, Spritz RA, Hearing VJ (2001). The molecular basis of oculocutaneous albinism type 1 (OCA1): sorting failure and degradation of mutant tyrosinases results in a lack of pigmentation. Biochem. J..

[47] Chaki M (2011). Molecular and functional studies of tyrosinase variants among Indian oculocutaneous albinism type 1 patients. J. Invest. Dermatol.

[48] Wei AH, Zang DJ, Zhang Z, Yang XM, Li W (2015). Prenatal genotyping of four common oculocutaneous albinism genes in 51 Chinese families. J. Genet. Genomics.

[49] Sulem P (2008). Two newly identified genetic determinants of pigmentation in Europeans. Nat. Genet.

[50] Nan H, Kraft P, Hunter DJ, Han J (2009). Genetic variants in pigmentation genes, pigmentary phenotypes, and risk of skin cancer in Caucasians. Int. J. Cancer.

[51] Sulem P (2007). Genetic determinants of hair, eye and skin pigmentation in Europeans. Nat. Genet..

[52] Stokowski RP (2007). A genomewide association study of skin pigmentation in a South Asian population. Am. J. Hum. Genet..

[53] Hu HH (2011). Assessment of tyrosinase variants and skin cancer risk in a large cohort of French subjects. J. Dermatol. Sci..

[54] Bouchard B, Fuller BB, Vijayasaradhi S, Houghton AN (1989). Induction of pigmentation in mouse fibroblasts by expression of human tyrosinase cDNA. J. Exp. Med.

[55] K B, Purohit R (2013). Mutational analysis of TYR gene and its structural consequences in OCA1A. Gene.

[56] Kobayashi T, Hearing VJ (2007). Direct interaction of tyrosinase with Tyrp1 to form heterodimeric complexes in vivo. J. Cell Sci..

[57] Orlow SJ (1994). High-molecular-weight forms of tyrosinase and the tyrosinase-related proteins: evidence for a melanogenic complex. J. Invest. Dermatol..

[58] van Dijk EL, Jaszczyszyn Y, Naquin D, Thermes C (2018). The third revolution in sequencing technology. Trends Genet..

[59] Adams, D. R. et al. One-year pilot study on the effects of nitisinone on melanin in patients with OCA-1B. *JCI Insight*10.1172/jci.insight.124387 (2019). 10.1172/jci.insight.124387 PMC6413781 30674731

[60] Lee H, Scott J, Griffiths H, Self JE, Lotery A (2019). Oral levodopa rescues retinal morphology and visual function in a murine model of human albinism. Pigment Cell Melanoma Res..

[61] Rawlins LE (2019). An Amish founder variant consolidates disruption of CEP55 as a cause of hydranencephaly and renal dysplasia. Eur. J. Hum. Genet..

[62] Chaki M, Mukhopadhyay A, Ray K (2005). Determination of variants in the 3’-region of the tyrosinase gene requires locus specific amplification. Hum. Mutat..

[63] Halaban R, Cheng E, Hebert DN (2002). Coexpression of wild-type tyrosinase enhances maturation of temperature-sensitive tyrosinase mutants. J. Invest. Dermatol.

[64] R Core Team. *R: A language and environment for statistical computing*. (Vienna, Austria, 2013). https://www.r-project.org/.

