# Supplementary material for: Evidence that the Ser192Tyr/Arg402Gln in cis Tyrosinase gene haplotype is a disease-causing allele in oculocutaneous albinism type 1B (OCA1B)
Source: NPJ Genom Med. 2022 Jan 13;7:2. doi: 10.1038/s41525-021-00275-9 (PMC8758782; doi:10.1038/s41525-021-00275-9)
Supplement: Supplementary file 1 — Supplementary Information [file 41525_2021_275_MOESM1_ESM.pdf]

Evidence that the Ser192Tyr/Arg402Gln in *cis* Tyrosinase gene haplotype is a disease-causing allele in oculocutaneous albinism type 1B (OCA1B)

Siying Lin[1], Aida Sanchez-Bretaño[2], Joseph S Leslie[1], Katie B Williams[3], Helena Lee[2,4], N Simon Thomas[5,6], Jonathan Callaway[5,6], James Deline[3], J Arjuna Ratnayaka[2], Diana Baralle[7], Melanie A Schmitt[8], Chelsea S Norman[2,9], Sheri Hammond[3], Gaurav V Harlalka[1,10], Sarah Ennis[11], Harold E Cross[12], Olivia Wenger[13,14], Andrew H Crosby[1 *] Emma L Baple[1,15 *], Jay E Self[2,4 *]

1. RILD Wellcome Wolfson Centre, Royal Devon & Exeter NHS Foundation Trust, Barrack Road, Exeter, UK
2. Clinical and Experimental Sciences, Faculty of Medicine, University of Southampton, Southampton, UK
3. Center for Special Children, Vernon Memorial Healthcare, La Farge, WI, USA
4. Southampton Eye Unit, University Hospital Southampton NHS Foundation Trust, Southampton, UK
5. Faculty of Medicine, University of Southampton, Southampton, UK
6. Wessex Regional Genetics Laboratory, Salisbury District Hospital, Salisbury, UK
7. Human Development and Health, Faculty of Medicine, University of Southampton, Southampton, UK
8. University of Wisconsin School of Medicine and Public Health, Department of Ophthalmology & Visual Sciences, Madison, WI, USA
9. The Rosalind Franklin Institute, Rutherford Appleton Laboratories, Harwell Science and Innovation Campus, Didcot, UK
10. Rajarshi Shahu College of Pharmacy, Malvihir, Buldana, India

11. Department of Human Genetics and Genomic Medicine, University of Southampton, Southampton, UK
12. Department of Ophthalmology, University of Arizona College of Medicine, Tucson, AZ, USA
13. New Leaf Clinic, PO Box 336, 16014 East Chestnut Street, Mount Eaton, OH, 44691, USA
14. Department of Pediatrics, Akron Children's Hospital, 214 West Bowery Street, Akron, OH 44308, USA
15. Peninsula Clinical Genetics Service, Royal Devon & Exeter Hospital (Heavitree), Gladstone Road, Exeter, UK

<u>Supplemental Table 1: Review of individuals homozygous for both *TYR* p.(Ser192Tyr) and p.(Arg402Gln)</u>

Individuals with a molecular diagnosis responsible for the albinism phenotype were excluded from this review.

Study	Cohort	No of individuals homozygous for both *TYR* p.(Ser192Tyr) and p.(Arg402Gln)	Comments
**Individuals with albinism with no alternative molecular diagnosis and homozygous for p.(Ser192Tyr)/p.(Arg402Gln)**			
Gronskov 2019[1]	93 individuals with a clinical diagnosis of albinism; diagnostic criteria included nystagmus, reduced visual acuity, iris translucency, fundus hypopigmentation, and foveal hypoplasia	6 individuals<br>• 5 with clinical diagnosis of AROA<br>• 1 with clinical diagnosis of OCA	• All 6 individuals were first investigated by sequential Sanger sequencing of 6 genes only (*TYR*, *OCA2*, *TYRP1*, *SLC45A2*, *LRMDA*, *GPR143*)<br>• 3 of the 6 individuals were further investigated by whole genome sequencing and data analysis of *TYR* genomic region
Campbell 2019[2]	12 individuals with nystagmus, at least one other ocular feature of albinism, and no apparent skin hypopigmentation in the context of their family	2 individuals (Proband 7 and 8; not related)	Proband 7<br>• Genomic data analysis limited to nystagmus and foveal hypoplasia gene panel (26 genes evaluated)<br>• Clinical information: iris transillumination, foveal hypoplasia, fundal hypopigmentation, probable VEP crossed asymmetry
			Proband 8<br>• Genomic data analysis limited to ocular/oculocutaneous albinism gene panel (18 genes evaluated)<br>• Rare, likely pathogenic *TYRP1* variant [c.208 G>A, p.(Ala70Thr)] also identified in this individual<br>• Affected brother with nystagmus, who was homozygous for both *TYR* p.(Ser192Tyr) and p.(Arg402Gln) but WT for the *TYRP1* variant

			• Clinical information: iris transillumination, foveal hypoplasia, fundal hypopigmentation, VEP not performed
This study	Salisbury cohort:<br>130 individuals with a clinical diagnosis of nystagmus and/or albinism<br><br>All were investigated with Illumina TruSight One clinical exome sequencing followed by additional filtering using virtual gene panel analysis using the "Albinism or congenital nystagmus v1.0" Panelpp gene panel	5 individuals	W1703356<br>• Foveal hypoplasia noted on OCT<br>• No other *TYR* or albinism-associated variant identified
			W1919237<br>• Afoveate adult with cystic fibrosis and nyctalopia<br>• No other *TYR* or albinism-associated variant identified<br>• Biallelic *ABCA4* variants also identified and further investigation with ERG planned; however foveal hypoplasia is not a known feature of *ABCA4*-associated retinal dystrophy
			W2002293<br>• Possibly afoveate (poor quality OCT scans)<br>• No other *TYR* variant or albinism-associated variant identified<br>• Maternal uncle apparently affected with nystagmus
			W1905299<br>• Afoveate, pale skin and hair, good albinism phenotype<br>• No other *TYR* variant or albinism-associated variant identified
			W1817121<br>• Afoveate; sister also afoveate but with no nystagmus<br>• No other *TYR* variant or albinism-associated variant identified

**Unaffected individuals homozygous for p.(Ser192Tyr)/p.(Arg402Gln)**			
Jagirdar 2014[3]	2 genetic epidemiological longitudinal studies: Brisbane Twin Nevus Study (1155 nuclear families) and Queensland Familial Melanoma Project (1211 melanoma cases)	2 individuals	Both individuals fair skinned with fair/blond hair and blue eye colour, no clinical diagnosis of OCA (but ocular examination not performed)
Campbell 2019[2]	100,000 genomes pilot dataset of 4046 individuals with no clinical features suggestive of albinism	2 individuals	No detailed ocular phenotyping available
This study	Southampton cohort:<br>161 probands with nystagmus and/or albinism and relatives	1 individual	This apparently unaffected individual was father to two affected siblings who were both clinically diagnosed with nystagmus and/or OCA (see below). Both affected siblings were heterozygous for a known pathogenic *TYR* variant, and also homozygous for both *TYR* p.(Ser192Tyr) and p.(Arg402Gln). This unaffected parent was asymptomatic but was noted to have with mild iris transillumination and foveal hypoplasia on OCT (see supplemental figure S2) in the absence of nystagmus or a pigmentary phenotype, and had normal visual acuities of 0.1 and 0.08 LogMAR (right and left eye respectively).
This study	Amish Exome Database:<br>Control exome database of 219 Amish individuals unaffected by OCA	2 individuals	No detailed ocular phenotyping available
**Individuals with albinism likely due to heterozygous deleterious *TYR* variant in combination with homozygosity for p.(Ser192Tyr)/p.(Arg402Gln)**			
This study	Southampton cohort:<br>161 probands with nystagmus and/or albinism and relatives	2 individuals	Affected siblings, both also heterozygous for a known pathogenic *TYR* variant
This study	Salisbury cohort:<br>130 individuals with a clinical diagnosis of nystagmus and/or albinism	1 individual	W1809902<br>Also heterozygous for a *TYR* pathogenic variant
Lasseaux 2018[4]	990 index patients with at least one of the main characteristic ocular features of albinism - either nystagmus or an absence of the fovea	1 individual	TYR/R402Q-P13<br>Also heterozygous for a known pathogenic *TYR* c.1118C>A; p.(Thr373Lys) variant[5]

Abbreviations: AROA, autosomal recessive ocular albinism; ERG, electroretinogram; OCA, oculocutaneous albinism; OCT, optical coherence tomography; VEP, visual evoked potential; WT, wild type

Supplemental Figure 1

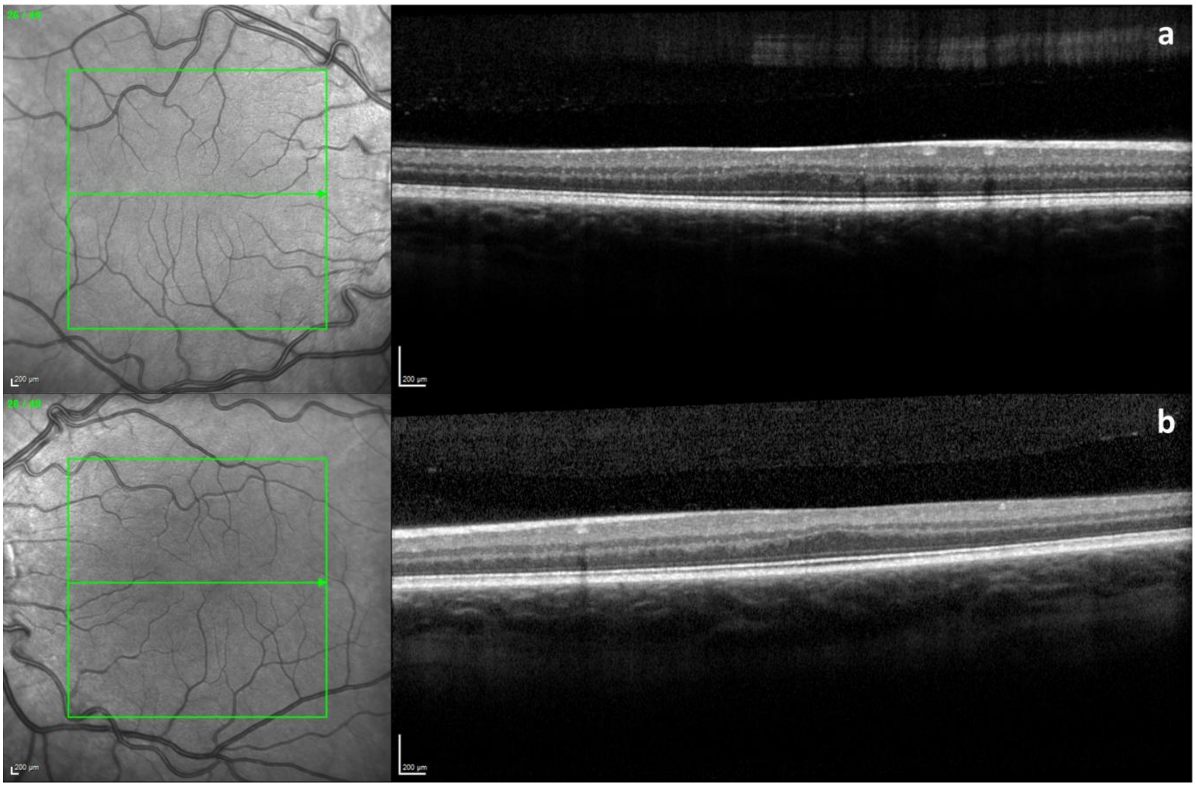

SD-OCT (Spectral domain optical coherence tomography; Spectralis-OCT, Heidelberg Engineering, Heidelberg, Germany) image of right (a) and left (b) eyes showing grade 3 foveal hypoplasia in an apparently unaffected individual homozygous for both *TYR* p.(Ser192Tyr) and p.(Arg402Gln) variants (foveal hypoplasia graded following structural grading system based on OCT data proposed by Thomas *et al* [6]).

Supplemental Table 2

Primers used to introduce each sequence mutation within the p3XFLAG-TYR vector. Underlined and bold letters indicate the base mutated in both variants, S192Y and R402Q, respectively.

Nucleotide Mutation	Amino Acid Change	Forward Primer (5'- 3')	Reverse Primer (5'- 3')
c.575A>C	Tyr192Ser	CTGCTTGGGGGAT**C**TGAGATCTGGAGAGACATTGATTTT	AATGTCTCTCCAGATCTCA**G**ATCCCCCAAGCAGTGCATCC
c.1205G>A	Arg402Gln	TTTGAACAGTGGCTCC**A**AAGGCACCGTCCTCTTCAAGAAG	TGAAGAGGACGGTGCCTT**T**GGAGCCACTGTTCAAAAATAC

Supplemental Table 3

Primer pairs used for sequencing the TYR coding exons and associated intron-exon junctions.

Primer	Primer Sequence 5' → 3'	Annealing temp (°C)	Amplicon size (bp)
TYR_Exon 1_F	TCAGCCAAGACATGTGATAATCA	60	992
TYR_Exon 1_R	TTATACCCTGCCTGAAGAAGTG		
TYR_Exon 2_F	CAACATTTCTGCCTTCTCCTA	55	888
TYR_Exon 2_R	CTGCCTAGAATATTTTAAACAGG		
TYR_Exon 3_F	GAATGAACAGGAGGGAACAC	58	470
TYR_Exon 3_R	TCTATTTAAATCCAATGAGCACGTT		
TYR_Exon 4_F	TTCTGGAGGTTCAAAACTCAATG	58	675
TYR_Exon 4_R	ACAAAATGGCCTATGTTAAGCAA		
TYR_Exon 5_F	TGTCTACTCCAAAGGACTGT	54	921
TYR_Exon 5_R	GGCACTTAGCTGGATGTGTT		

REFERENCES

1 Gronskov, K. *et al.* A pathogenic haplotype, common in Europeans, causes autosomal recessive albinism and uncovers missing heritability in OCA1. *Sci Rep* **9**, 645, doi:10.1038/s41598-018-37272-5 (2019).

2 Campbell, P. *et al.* Clinical and genetic variability in children with partial albinism. *Sci Rep* **9**, 16576, doi:10.1038/s41598-019-51768-8 (2019).

3 Jagirdar, K. *et al.* Molecular analysis of common polymorphisms within the human Tyrosinase locus and genetic association with pigmentation traits. *Pigment Cell Melanoma Res* **27**, 552-564, doi:10.1111/pcmr.12253 (2014).

4 Lasseaux, E. *et al.* Molecular characterization of a series of 990 index patients with albinism. *Pigment Cell Melanoma Res* **31**, 466-474, doi:10.1111/pcmr.12688 (2018).

5 Tripathi, R. K., Hearing, V. J., Urabe, K., Aroca, P. & Spritz, R. A. Mutational mapping of the catalytic activities of human tyrosinase. *J Biol Chem* **267**, 23707-23712 (1992).

6 Thomas, M. G. *et al.* Structural grading of foveal hypoplasia using spectral-domain optical coherence tomography: a predictor of visual acuity? *Ophthalmology* **118**, 1653-1660 (2011).
