# Supplementary material for: Evidence that the Ser192Tyr/Arg402Gln in cis Tyrosinase gene haplotype is a disease-causing allele in oculocutaneous albinism type 1B (OCA1B)
Source: NPJ Genom Med. 2022 Jan 13;7:2. doi: 10.1038/s41525-021-00275-9 (PMC8758782; doi:10.1038/s41525-021-00275-9)
Supplement: Supplementary file 2 — Reporting Summary [file 41525_2021_275_MOESM2_ESM.pdf]

nature portfolio

Corresponding author(s): Dr James E Self
Dr Emma L Baple
Professor Andrew H Crosby

Last updated by author(s): Nov 3, 2021

Reporting Summary

Nature Portfolio wishes to improve the reproducibility of the work that we publish. This form provides structure for consistency and transparency in reporting. For further information on Nature Portfolio policies, see our Editorial Policies and the Editorial Policy Checklist.

Statistics

For all statistical analyses, confirm that the following items are present in the figure legend, table legend, main text, or Methods section.

n/a	Confirmed	
☐	☒	The exact sample size (n) for each experimental group/condition, given as a discrete number and unit of measurement
☐	☒	A statement on whether measurements were taken from distinct samples or whether the same sample was measured repeatedly
☐	☒	The statistical test(s) used AND whether they are one- or two-sided<br>*Only common tests should be described solely by name; describe more complex techniques in the Methods section.*
☐	☒	A description of all covariates tested
☐	☒	A description of any assumptions or corrections, such as tests of normality and adjustment for multiple comparisons
☐	☒	A full description of the statistical parameters including central tendency (e.g. means) or other basic estimates (e.g. regression coefficient) AND variation (e.g. standard deviation) or associated estimates of uncertainty (e.g. confidence intervals)
☐	☒	For null hypothesis testing, the test statistic (e.g. F, t, r) with confidence intervals, effect sizes, degrees of freedom and P value noted<br>*Give P values as exact values whenever suitable.*
☒	☐	For Bayesian analysis, information on the choice of priors and Markov chain Monte Carlo settings
☒	☐	For hierarchical and complex designs, identification of the appropriate level for tests and full reporting of outcomes
☒	☐	Estimates of effect sizes (e.g. Cohen's d, Pearson's r), indicating how they were calculated

Our web collection on statistics for biologists contains articles on many of the points above.

Software and code

Policy information about availability of computer code

Data collection	Statistical analysis was performed using established software packages (R and SPSS)
Data analysis	Statistical analysis was performed using established software packages (R and SPSS)

For manuscripts utilizing custom algorithms or software that are central to the research but not yet described in published literature, software must be made available to editors and reviewers. We strongly encourage code deposition in a community repository (e.g. GitHub). See the Nature Portfolio guidelines for submitting code & software for further information.

Data

Policy information about availability of data

All manuscripts must include a data availability statement. This statement should provide the following information, where applicable:

- Accession codes, unique identifiers, or web links for publicly available datasets
- A description of any restrictions on data availability
- For clinical datasets or third party data, please ensure that the statement adheres to our policy

The datasets generated and/or analysed during the current study are available from the corresponding author upon reasonable request.

Field-specific reporting

Please select the one below that is the best fit for your research. If you are not sure, read the appropriate sections before making your selection.

☒ Life sciences ☐ Behavioural & social sciences ☐ Ecological, evolutionary & environmental sciences

For a reference copy of the document with all sections, see nature.com/documents/nr-reporting-summary-flat.pdf

Life sciences study design

All studies must disclose on these points even when the disclosure is negative.

Sample size	A clinical cohort of affected individuals with nystagmus and/or albinism was retrospectively ascertained through the Southampton (161 individuals) and Salisbury (131 individuals) research databases.<br><br>An additional 37 affected individuals and unaffected family members from four Ohio and Wisconsin Amish families were recruited to this study.
Data exclusions	All recruited Amish family members, and all eligible individuals ascertained retrospectively through the Southampton and Salisbury research databases were included in the study.
Replication	Enzymatic assays were routinely performed in triplicate and the results are presented as the means of the independent assays +/- standard error.
Randomization	Not relevant as study is not a randomised controlled trial
Blinding	Not relevant as study is not a randomised controlled trial

Reporting for specific materials, systems and methods

We require information from authors about some types of materials, experimental systems and methods used in many studies. Here, indicate whether each material, system or method listed is relevant to your study. If you are not sure if a list item applies to your research, read the appropriate section before selecting a response.

Materials & experimental systems

n/a	Involved in the study	
☒	☐	Antibodies
☐	☒	Eukaryotic cell lines
☒	☐	Palaeontology and archaeology
☒	☐	Animals and other organisms
☐	☒	Human research participants
☒	☐	Clinical data
☒	☐	Dual use research of concern

Methods

n/a	Involved in the study	
☒	☐	ChIP-seq
☒	☐	Flow cytometry
☒	☐	MRI-based neuroimaging

Eukaryotic cell lines

Policy information about cell lines

Cell line source(s)	Human Embryonic Kidney 293 Freestyle (HEK293F) cells (Invitrogen, California, USA)
Authentication	Cell lines not authenticated
Mycoplasma contamination	No mycoplasma testing done
Commonly misidentified lines (See ICLAC register)	No commonly misidentified similar lines

Human research participants

Policy information about studies involving human research participants

Population characteristics	Affected individuals and unaffected family members from four Ohio and Wisconsin Amish families with a common Ohio ancestry were recruited to this study.<br><br>Additionally, a UK clinical cohort of affected individuals with nystagmus and/or albinism was retrospectively ascertained

	through the Southampton (161 individuals) and Salisbury (131 individuals) research databases. All individuals had been referred from a regional paediatric nystagmus clinic and offered recruitment.
Recruitment	All available affected and unaffected family members from the four Ohio and Wisconsin Amish families were offered recruitment.<br><br>Individuals in the Southampton and Salisbury research databases had been all been referred from a regional paediatric nystagmus clinic and offered recruitment.
Ethics oversight	University of Arizona IRB—1000000050<br>Akron Children's Hospital IRB – project number 986876-3<br>South Central – Hampshire A Research Ethics Committee – IRAS:174564

Note that full information on the approval of the study protocol must also be provided in the manuscript.
